# Identification and validation of selenium metabolism-related genes in lung adenocarcinoma prognosis using bioinformatics analysis

**DOI:** 10.3389/fgene.2025.1655262

**Published:** 2025-10-16

**Authors:** Yun Chen, Ping Li, Yang Wang, Shuai Shen, Ni Chen, Hao Peng, Zheyuan Xu

**Affiliations:** ^1^ Department of Thoracic Surgery, the First People’s Hospital of Yunnan Province, The Affiliated Hospital of Kunming University of Science and Technology, Kunming, China; ^2^ Department of Cardiovascular Medicine, The First People’s Hospital of Kunming City & Ganmei Affiliated Hospital of Kunming Medical University, Kunming, China

**Keywords:** lung adenocarcinoma, selenium metabolism, prognostic model, tumor microenvironment, immunotherapy response, drug sensitivity

## Abstract

**Background:**

The disruption of selenium metabolism has been associated with tumor progression. However, the prognostic significance and underlying molecular mechanisms of selenium metabolism in lung adenocarcinoma (LUAD) remain inadequately understood. This study primarily aimed to identify and validate prognostic genes related to selenium metabolism in LUAD patients.

**Methods:**

Transcriptomic datasets from patients diagnosed with LUAD were meticulously analyzed to identify differentially expressed genes associated with selenium metabolism. The genes selected for the prognostic risk model were determined through various analyses, including differential gene expression assessment, univariate and multivariate Cox proportional hazards regression analyses, as well as other relevant analytical methods. A systematic approach was employed for functional enrichment analysis, characterization of the immune microenvironment, somatic mutation analysis, and evaluation of drug sensitivity to elucidate the mechanisms linked to prognostic genes and risk categories. Finally, a reverse transcription quantitative PCR(RT-qPCR) assay was conducted to validate the expression levels of the identified prognostic genes.

**Results:**

F2, GPX3, KMO, and KYNU were identified as prognostic genes for establishing a risk model. The functions of these LUAD prognostic genes were influenced by DNA replication pathways, cell cycle regulation, and quiescent CD4 memory T cells. In the high-risk group (HRG), KEAP1, TTN, and USH2A exhibited the highest mutation rate at 48%, while TTN had an even higher mutation rate of 52% in the low-risk group (LRG). Within the HRG cohort, both cisplatin and gemcitabine demonstrated significant sensitivity. Ultimately, RT-qPCR findings corroborated results obtained from bioinformatics analyses; specifically compared to normal samples: GPX3, KMO, KYNU showed significant downregulation in LUAD tissues while F2 was found to be upregulated in LUAD.

**Conclusion:**

This study identified four prognostic genes in LUAD and examined their associated mechanisms of action, which may contribute to the development of novel treatment strategies. The integration of immune characterization with drug sensitivity analysis offers valuable insights for stratified therapy.

## 1 Introduction

Lung cancer is one of the most prevalent forms of cancer worldwide, with lung adenocarcinoma (LUAD) being the most commonly diagnosed subtype (Huang et al., 2025). This disease represents a significant threat to human health, accounting for over 700,000 deaths annually. The high incidence and mortality rates associated with LUAD have made it a critical focus for global public health initiatives. Despite advancements in early detection methods and the continuous evolution of treatment modalities, including surgery, radiotherapy, chemotherapy, targeted therapy, and immunotherapy, the overall 5-year survival rate remains alarmingly low at approximately 25%. This is primarily due to elevated rates of recurrence and metastasis. Therefore, identifying new prognostic biomarkers and therapeutic targets is essential for improving patient outcomes in lung cancer (Goldberg and Herbst, 2023). In recent years, rapid progress in genomics and bioinformatics has enabled researchers to explore the relationship between gene expression patterns and lung cancer prognosis (Dizon and Kamal, 2024). By comparing gene expression profiles from tumor tissues with those from normal tissues, several genes associated with LUAD prognosis have been identified. Variations in their expression levels reflect both the biological behavior of the tumor and its clinical outcomes. A deeper understanding of these genes’ functions as well as their roles in LUAD progression can facilitate the development of novel treatment strategies aimed at enhancing survival rates. Furthermore, studies indicate that the immune status of LUAD patients is closely linked to the degree of immune infiltration within tumors (Zhang et al., 2022), which may influence tumor growth and metastasis (Zhang et al., 2021). Consequently, characterizing the immune microenvironment of LUAD, particularly regarding the distribution and functional roles of immune cells within tumors, holds great promise for designing personalized treatment plans. These studies underscore the significance of public datasets and bioinformatics tools in the identification of clinically relevant molecular targets.

Selenium is a crucial trace element that plays an integral role in human metabolism, particularly within the antioxidant defense system (Zhang et al., 2023). Selenium metabolism facilitates the elimination of free radicals by forming the active center of glutathione peroxidase, thereby safeguarding cells against oxidative damage (Sun et al., 2023). Furthermore, selenium is closely linked to immune function and enhances the body’s capacity to resist diseases (Luo et al., 2025). In cancer research, adequate selenium intake has been associated with a reduced risk of certain types of cancer. Consequently, elucidating the role and impact of selenium metabolism in cancer biology may yield new insights into prevention and treatment strategies. In the context of LUAD the anti-tumor effects of selenium have attracted increasing attention. Clinical and laboratory studies indicate that selenium deficiency may correlate with a heightened risk of tumor development, while sufficient selenium intake could inhibit tumor cell proliferation and improve patient outcomes (Liang et al., 2024). Moreover, the anti-tumor effects attributed to selenium metabolism may modulate multiple signaling pathways such as PI3K/Akt and MAPK, influencing processes related to cancer cell proliferation, apoptosis, and migration (Guo et al., 2022; Xiao et al., 2025). Therefore, investigating the precise mechanisms underlying selenium metabolism in LUAD could open new avenues for early diagnosis and personalized treatment strategies, holding significant scientific and clinical implications.

In summary, while there has been notable progress in the treatment of LUAD, challenges persist regarding early diagnosis and prognosis. Gene expression and the immune microenvironment are critical factors influencing tumor initiation and progression. Selenium metabolism shows promise in modulating tumor development through mechanisms such as oxidative stress regulation and immune modulation. Investigating these elements may facilitate the identification of new prognostic biomarkers and therapeutic targets. This study primarily utilized transcriptome data from public databases pertaining to LUAD patients, employing bioinformatics techniques to construct and validate a novel selenium metabolism-related risk model aimed at predicting early recurrence in LUAD patients. Additionally, we analyzed the biological pathways associated with these genes, along with their relationships to clinical characteristics, somatic mutations, immune microenvironment dynamics, immunotherapy responses, and drug sensitivity. The expression levels of these prognosis-related genes were validated using clinical samples, thereby supporting the advancement of new chemotherapy regimens, immunotherapies, and targeted therapies for LUAD.

## 2 Materials and methods

### 2.1 Data acquisition and preprocessing

A transcriptomic dataset was obtained from The Cancer Genome Atlas (TCGA) database (https://portal.gdc.cancer.gov/, accessed on 15 January 2025), which includes RNA sequencing data along with clinical and pathological information for 530 LUAD samples and 59 normal samples (TCGA-LUAD) (Liu et al., 2019). Among these, a total of 517 LUAD samples contained complete survival data, while 222 samples had comprehensive clinical characteristics documented. An independent validation cohort, GSE26939, was retrieved from the Gene Expression Omnibus (GEO) database (https://www.ncbi.nlm.nih.gov/geo/), comprising 116 LUAD samples, 115 of which included survival information, profiled using the GPL9053 expression profiling by array platform (Song et al., 2022). Furthermore, a curated list of 86 selenium metabolism-related genes (SMRGs) was compiled based on published literature (Fu et al., 2023).

### 2.2 Investigation of differential gene expression patterns in TCGA-LUAD

Differentially expressed genes (DEGs) that distinguish LUAD from non-tumor specimens within the TCGA-LUAD dataset were identified utilizing the R package “DESeq2” (v 1.4.2) (Love et al., 2014). For the downloaded raw data, it was recalibrated into a count matrix and filtered to retain genes exhibiting a total expression level greater than ten across all samples, applying criteria of adjusted p < 0.05 and absolute log2-fold change (FC) > 1. Visualization was conducted using the R package “ggVolcano” (v 0.0.2) (Wodrich et al., 2021), resulting in the generation of a volcano plot. Additionally, heatmap visualization was performed employing the R package “ComplexHeatmap” (v 2.14.0) (Gu et al., 2016).

### 2.3 Identifying candidate genes and analyzing their functions

To identify candidate genes related to selenium metabolism, we utilized the R package “VennDiagram” (version 1.7.3) (Chen and Boutros, 2011) to visualize the intersection analysis between DEGs and SMRGs, generating a Venn diagram. Subsequently, a functional enrichment assay was performed to investigate the biological roles of these candidate genes. Gene Ontology (GO) analysis was conducted using the enrichGO function via the “clusterProfiler” package (version 4.2.2) (Wu et al., 2021), employing the “SYMBOL” gene identifier sourced from the “org.Hs.e.g.,.db” annotation database (version 3.18.0) (Qing et al., 2022) (adjusted p < 0.05). Furthermore, for Kyoto Encyclopedia of Genes and Genomes (KEGG) enrichment analysis, we applied the enrichKEGG function within the same R package (“clusterProfiler”), focusing on human genome data with organism set as “hsa,” while maintaining identical significance thresholds of adjusted p < 0.05.

### 2.4 Protein-protein interaction (PPI) network analysis

The STRING database was used to build PPI networks to investigate the functional relationships among candidate genes (confidence >0.4). Following this, Cytoscape software version 3.8.2 (Smoot et al., 2011) was utilized for visualizing these interaction networks.

### 2.5 Development, assessment, and verification of the risk score model

Utilizing the “survival” package (version 3.7.0) (Lei et al., 2023), a univariate Cox regression analysis was conducted on LUAD samples with comprehensive survival data, using candidate genes as the basis for evaluation. Genes that met significance thresholds (HR ≠ 1, p < 0.05) and satisfied the proportional hazards (PH) assumption, assessed via the cox. zph function from the “survival” package, were retained (p > 0.05). Subsequently, we employed the “survival” package to construct a multivariable Cox regression model utilizing its built-in functions for Cox regression analysis. A bidirectional stepwise variable selection process was implemented based on the Akaike Information Criterion (AIC), employing the step function; additionally, an overall PH assumption test of the model was performed (p > 0.05). Through this methodology, prognostic genes were identified. Risk scores were then calculated by applying coefficients derived from this final model to determine risk associated with each prognostic gene in constructing our risk model. In this context, “coef” refers to the risk coefficient corresponding to each individual prognostic gene, while “expr” indicates the expression intensity of each respective prognostic gene.

$$\text{risk score}=\sum_{i=1}^{n}\text{coef}\left({\text{gene}}_{i}\right)\times \text{expr}\left({\text{gene}}_{i}\right)$$

The optimal cutoff value derived from the surv_cutpoint function in the R package “survminer” (version 0.4.9) (Liu et al., 2021) was utilized to classify patients into high-risk groups (HRG) and low-risk groups (LRG). Subsequently, principal component analysis (PCA) was performed using the prcomp function to visualize the separation of risk groups. Kaplan-Meier (KM) survival plots and log-rank tests, facilitated by the R package “survminer,” were employed to compare survival outcomes between these groups. The software package “timeROC” (version 1.18.0) (Blanche et al., 2013) was leveraged to generate receiver operating characteristic (ROC) curves at three time points for evaluating model precision. Following this, expression levels of prognostic genes across both cohorts were examined utilizing the “pheatmap” package (version 1.0.12) (Gu and Hübschmann, 2022). For external validation, data from GSE26939 were analyzed with identical risk stratification methods to assess the robustness of the model.

### 2.6 Development and validation of the nomogram

A univariate Cox regression analysis was conducted on risk scores alongside several clinical variables, including pathological stages, gender, age, and T/N/M stages (p < 0.05, HR ≠ 1); additionally, a proportional hazards (PH) assumption test was performed with p > 0.05 as an acceptance criterion. Variables that met criteria in multivariate Cox regression analysis, specifically those with p < 0.05, and passed PH assumption testing were considered independent predictive factors for prognosis. Based on TCGA-LUAD data, we developed a nomogram to forecast 1-, 3-, and 5-year survival probabilities with the “regplot” package (version 1.1) (Sui et al., 2022). Thereafter, we generated a calibration curve employing bootstrap resampling techniques through two hundred iterations via the R package “rms” (version 6.8.1) (Sachs, 2017), aimed at assessing nomogram accuracy; proximity of slope values to one indicates enhanced predictive accuracy of our model’s estimates for patient outcomes over time.

Furthermore, we utilized the R package “ggDCA” version v (1.1) (Duo et al., 2023) to create a comprehensive nomogram forecasting 1-,3-,and 5-year survival probabilities for patients diagnosed with LUAD.

### 2.7 Examination of the relationship between risk scores and the expression of prognostic genes within clinical variables

To investigate the diagnostic efficiency of risk scores in relation to clinical indicators, we analyzed correlations between risk scores and the aforementioned clinical variables. The Wilcoxon test (W-tests) was employed to compare varying risk scores and prognostic genes across different clinical subgroups (p < 0.05). Additionally, the survdiff function within the “survival” package was employed to assess survival differences among risk groups for each clinical indicator (p < 0.05).

### 2.8 Functional enrichment analysis

Within the TCGA-LUAD data collection, Gene Set Enrichment Analysis (GSEA) was conducted to pinpoint biological pathways linked to HRG and LRG. Initially, differential gene expression analysis was performed between the two risk groups via the “DESeq2″ package (v 1.4.2), resulting in a ranked list of genes ordered by their log_2_FC values from largest to smallest. Subsequently, we retrieved a background gene set named “c2. cp.kegg.v7.4. symbols.gmt” from the MSigDB for pathway enrichment analysis utilizing the “clusterProfiler” package (v 4.2.2). Significant pathways were defined as those exhibiting a normalized enrichment score (|NES|) > 1, p < 0.05, and false discovery rate (FDR) < 0.25.

For Gene Set Variation Analysis (GSVA), we employed the “GSVA” package (v 1.42.0) (Hänzelmann et al., 2013) to calculate pathway activity scores across samples denoted as gsva_mat. A design matrix was constructed using the model. matrix function from the “limma” package (v 3.54.0) (Ritchie et al., 2015), followed by an assessment of differential pathway activity between risk groups through linear model fitting via lmFit and empirical Bayes moderation using eBayes methods. Contrast matrices were generated with makeContrasts function, allowing us to extract significantly altered pathways with adjusted p-values <0.05 through topTable function.

### 2.9 Analysis of the tumor immune microenvironment

The CIBERSORT method was employed to estimate the proportions of 22 distinct immune cell subtypes (Newman et al., 2015) within the tumor microenvironment of LUAD samples. Gene expression matrices underwent preprocessing and were subsequently submitted to the CIBERSORT platform, with 10 permutations and quantile normalization (QN) activated. To compare the levels of immune cell infiltration among different risk groups, W-tests were conducted (p < 0.05). The “psych” package (v 2.1.6) (Correction to Lancet Psych, 2022, 2023) was utilized to calculate Spearman correlation coefficients between differentially abundant immune cells and risk scores, as well as between these immune cells and prognostic genes (|correlation coefficient (cor)| > 0.3, p < 0.05).

### 2.10 Analysis of LUAD immunotherapy response

The Tumor Immune Dysfunction and Exclusion (TIDE) platform was used to assess mechanisms of immune evasion and predict responses to immunotherapy. Pre-treatment gene expression profiles from LUAD samples were standardized before being submitted to TIDE for generating Dysfunction, Exclusion, and TIDE scores. Dysfunction scores reflect the functional state of effector T lymphocytes within the tumor microenvironment, while Exclusion scores indicate barriers to immune cell infiltration.

Furthermore, a total of 47 immune checkpoint genes (e.g., LAG-3, CTLA-4, PD-1) were extracted from relevant literature (Xue et al., 2022). A differential expression analysis on these genes across risk groups was performed using W-tests (p < 0.05).

### 2.11 Somatic mutation profiling

The TCGA database was queried to obtain somatic mutation information for LUAD samples. Subsequently, the “Maftools” package (v 2.18.0) (Mayakonda et al., 2018) was employed to analyze the mutation annotation format (MAF) files and generate oncoplots (waterfall plots) that illustrate the mutation landscape of HRG and LRG. Entries with missing values were excluded following the annotation and filtering of variant types and classifications. The distribution of mutation types and allele-specific changes was assessed. Thereafter, using the R package “ggplot2” (v 3.4.1) (Gustavsson et al., 2022), we visualized the distribution of these mutation types across HRG and LRG populations. Tumor mutational burden (TMB), defined as the cumulative count of somatic mutations per megabase, was calculated with statistical significance set at p < 0.05. Meanwhile, the chi-square test was used to analyze the differences in the top 10 mutation classifications, different mutation types, and base changes between HRG and LRG (p < 0.05). In addition, the somaticInteractions function in the “Maftools” package (v 2.18.0) was utilized to perform co - occurrence and mutual exclusivity analysis on the mutation data of the high - risk and low - risk groups (p < 0.05).

### 2.12 Drug sensitivity analyses in risk groups

Drug sensitivity analyses were conducted utilizing the R package “pRRophetic” (v 0.5) (Geeleher et al., 2014). This method used the Genomics of Drug Sensitivity in Cancer (GDSC, also known as CGP 2016) as the reference training set to construct a drug sensitivity prediction model. Specifically, we input the gene expression matrix (FPKM) of the TCGA-LUAD cohort into the model, with parameters set as tissueType = “lung” and dataset = “cgp 2016”. Subsequently, the pRRopheticPredict () function was applied to calculate the predicted half-maximal inhibitory concentration (IC50) of each drug for each patient. Initially, gene expression data from the TCGA database were integrated with a well-established drug sensitivity dataset known as cgp 2016; drugs classified under LUAD by TCGA with an area under curve (AUC) greater than 0.98 were selected as candidates for further analysis. The half-maximal inhibitory concentration (IC_50_) for each drug were calculated using the pRRopheticPredict function, followed by a W-test to identify disparities in IC_50_ values among various drugs between HRG and LRG cohorts, establishing statistical significance at p < 0.01. Additionally, we utilized the R software package “pheatmap” to visually represent relationships between first-line chemotherapeutic agents and prognostic genes in LUAD.

### 2.13 Validation of the expression of prognostic genes in clinical samples

Drawing on TCGA-LUAD data, a W-test was first conducted to evaluate the expression of prognostic genes in LUAD samples relative to normal samples (p < 0.05). Boxplots were created with the “ggplot2″ package.

Ethical approval was obtained from the ethics committee of the First People’s Hospital of Yunnan Province (approval number: KHLL2022-KY159) before this experiment was conducted. Five LUAD tissue samples and five adjacent non-tumor tissue samples were obtained from participants at the First People’s Hospital of Yunnan Province, all of whom provided informed consent. Initially, 50 mg of tissue was extracted from each sample and homogenized with 1 mL of TRIzol (Vazyme, R401-01, China) to ensure thorough mixing and grinding. After standing on ice for 10 min, 200 µL of chloroform was added to facilitate RNA extraction from the aqueous phase. Subsequently, an equal volume of chilled isopropanol was incorporated for RNA precipitation. Following quantification, reverse transcription reactions commenced immediately thereafter. The cDNA synthesis reaction system was established strictly according to the manufacturer’s instructions for the SweScript First Strand cDNA Synthesis Kit (YEASEN, 11141ES60, China). Thereafter, qPCR amplification consisting of 40 cycles was conducted using a CFX96 real-time fluorescence quantitative PCR device (BIO-RAD, XLFZ006, United States). The primer sequences are detailed in Supplementary Table S1. In this study, glyceraldehyde-3-phosphate dehydrogenase (GAPDH) was selected as the reference gene. In terms of experimental design, five biological replicates were set up to reduce the interference of individual sample differences, and three technical replicates were performed for each sample to ensure the repeatability of detection; after the completion of qPCR amplification, the cycle threshold (Ct values) of target genes and the reference gene were obtained simultaneously, and the melting curve was used to verify amplification specificity, while the amplification curve was used to evaluate amplification efficiency.

The expression levels of prognostic genes were evaluated utilizing the 2^−ΔΔCt^ method (Cheng et al., 2020), with specific steps as follows: first, the ΔCt value was calculated as the difference between the Ct value of the target gene and that of GAPDH (ΔCt = Ct_target gene - Ct_GAPDH); second, the ΔΔCt value was calculated by taking the ΔCt value of the control group as a reference and finding the difference between the ΔCt value of the experimental group and that of the control group (ΔΔCt = ΔCt_experimental group - ΔCt_control group);.finally, the relative expression level of the target gene was obtained through conversion using the 2^−ΔΔCt^ formula. All experimental data were subjected to statistical analysis and visualization using GraphPad Prism (v8.0) software (Chang et al., 2023): the t-test was used as the statistical method, and a p < 0.05 was considered to indicate a statistically significant difference.

### 2.14 Statistical analysis

All statistical analyses of data from public databases were performed using R software (version 4.2.2). The Wilcoxon test and chi-square test were used as the significance test method to compare differences between different groups, and a p < 0.05 was considered to indicate statistical significance. All network diagrams were constructed using Cytoscape software (v 3.8.2). For RT-qPCR experiments, the relative mRNA expression levels of prognostic genes were calculated using the 2^−ΔΔCt^ method, and the t-test was applied to compare differences in expression levels. A p < 0.05 was regarded as statistically significant.

## 3 Results

### 3.1 Differential expression profiling

**FIGURE 1:**
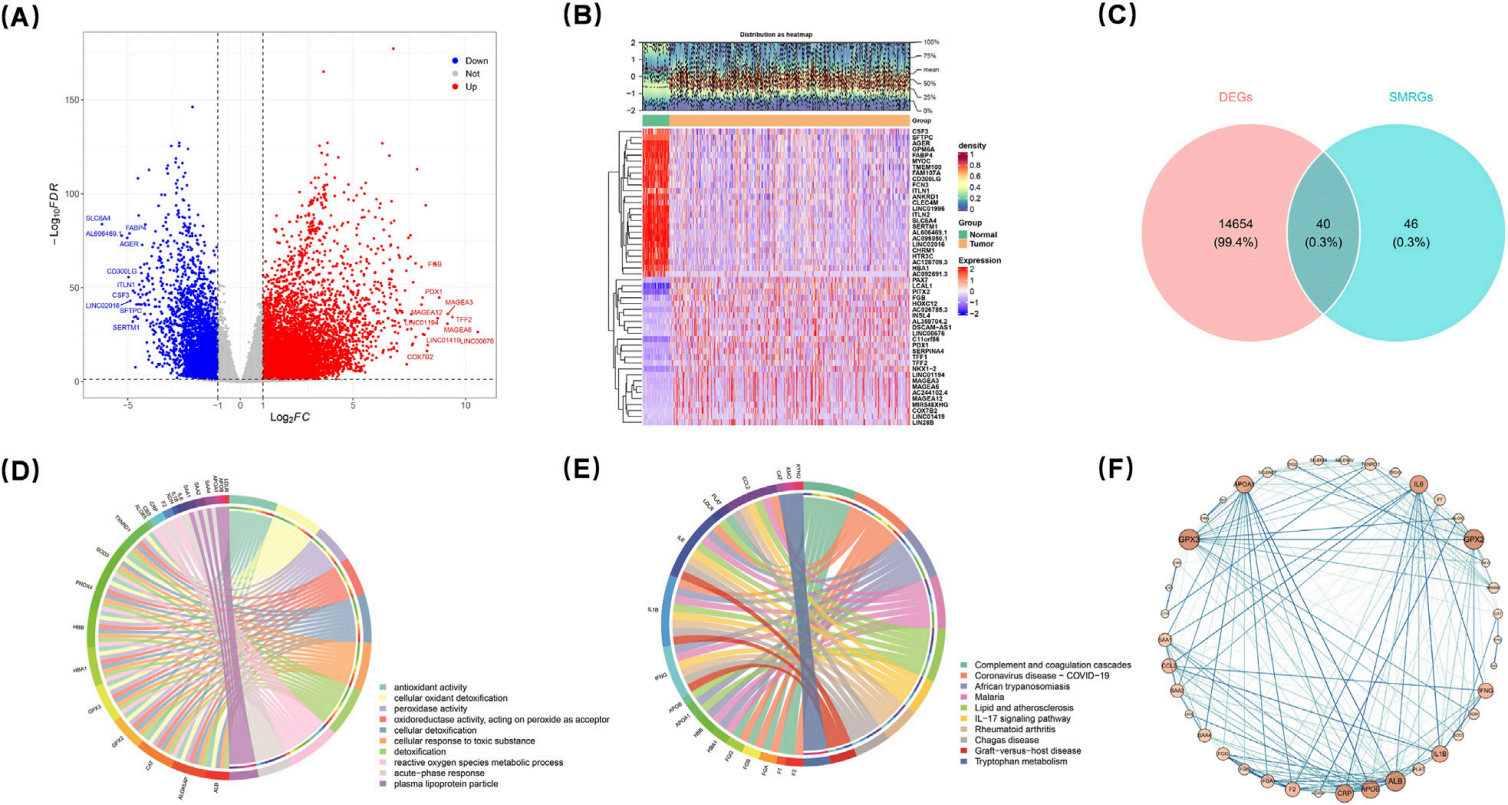
Comparative analysis identified differentially regulated genes, verified by GO, KEGG and PPI analyses. Comparative analysis of LUAD vs. normal tissues identified 14,694 DEGs, including 11,390 upregulated and 3,304 downregulated genes **(A)**. The top 50 DEGs were visualized in a heatmap **(B)**. Intersection analysis revealed 40 candidate genes **(C)**. GO analysis identified 504 significant entries, with antioxidant activity and oxidant detoxification as the most enriched **(D)**. KEGG analysis showed 29 enriched pathways, including complement/coagulation cascades and IL-17 signaling **(E)**. PPI network analysis demonstrated interactions among candidate gene-encoded proteins **(F)**.

Comparative analysis of LUAD versus normal tissues revealed a total of 14,694 DEGs (adjusted p < 0.05 and |log_2_FC| > 1), comprising 11,390 upregulated genes and 3,304 downregulated genes (Figure 1A). The top 50 DEGs exhibiting the largest |log_2_FC| values were visualized in a hierarchical clustering heatmap (Figure 1B), which highlighted distinct expression patterns between LUAD and normal tissue groups. Intersection analysis between DEGs and survival-related gene sets identified 40 candidate genes (Figure 1C) (Supplementary Table S2).

### 3.2 Functional enrichment characteristics

GO analysis of the candidate genes uncovered a total of 504 significant entries (adjusted p < 0.05), with antioxidant activity (GO:0016209, p = 1.35e-15) and cellular oxidant detoxification (GO:0098869, p = 8.44e-15) being the most enriched categories (Figure 1D) (Supplementary Table S3). Additionally, KEGG pathway analysis indicated significant enrichment across 29 pathways (adjusted p < 0.05). Notably among these pathways were the complement and coagulation cascades pathway (hsa04610, p = 4.83e-07) as well as the IL-17 signaling pathway (hsa04657, p = 0.000311) (Figure 1E) (Supplementary Table S4). Furthermore, PPI network analysis demonstrated interactions among proteins encoded by the identified candidate genes; specifically, ALB, GPX3, GPX2, IL6, APOB, APOA1, and CRP exhibited interactions with several other candidate gene products (Figure 1F). Collectively, this PPI network provides valuable insights into the dynamic interactions among proteins corresponding to these relevant genes at the protein level.

### 3.3 Development and validation of the prognostic model

**FIGURE 2:**
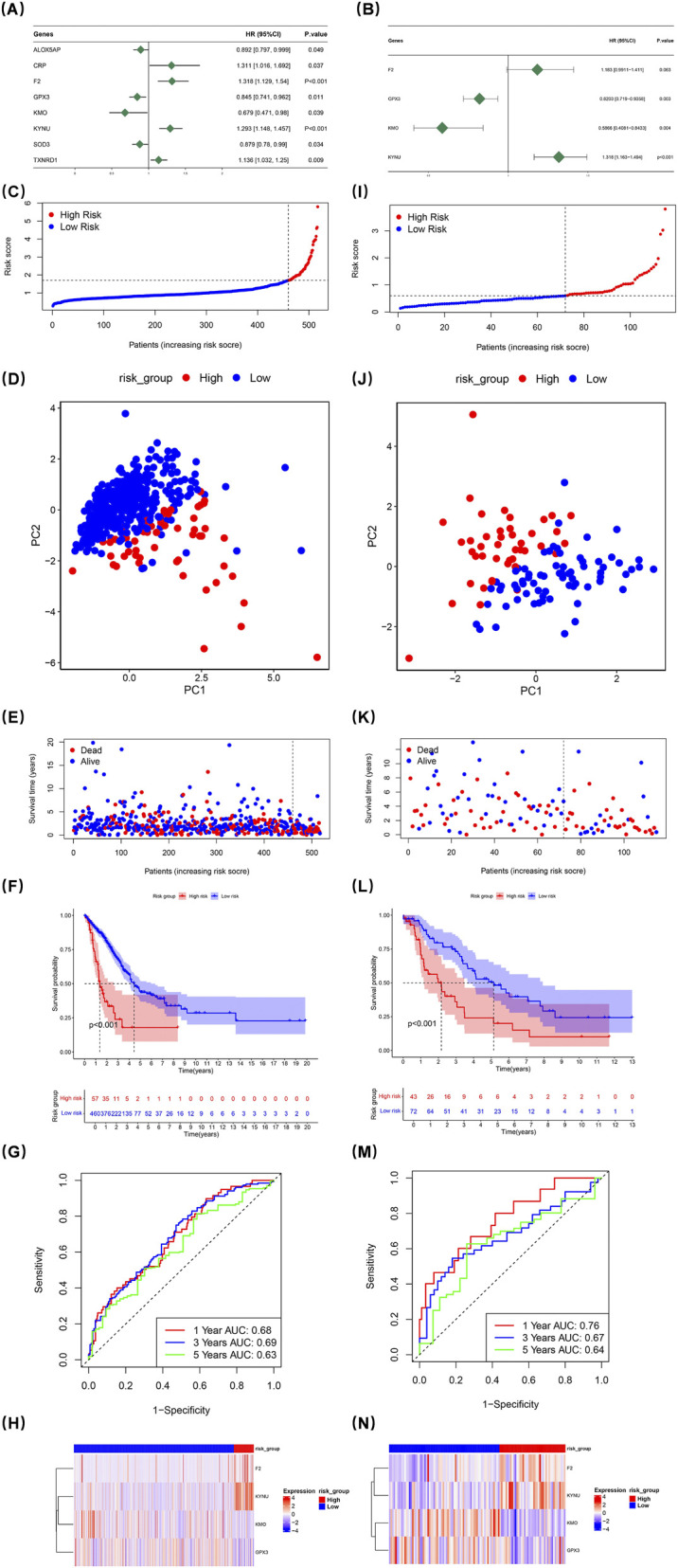
Univariate Cox regression analysis of 517 LUAD samples from TCGA-LUAD identified genes correlated with overall survival. ALOX5AP, CRP, F2, GPX3, KMO, KYNU, SOD3, and TXNRD1 were found as risk factors **(A)**. Schoenfeld residual plots confirmed proportional hazards (p > 0.05) (Supplementary Figure S1A–H). A multifactorial Cox regression model screened four prognostic genes: GPX3, KMO, KYNU, and TXNRD1 **(B)**. The model met the proportional hazards assumption (p = 0.228) (Supplementary Figure S1I) (Supplementary Table S5). Risk scores were calculated, classifying patients into high-risk (HRG) and low-risk (LRG) groups **(C, I)**. PCA analysis showed distinct separation between HRG and LRG **(D,J)**. Mortality rates were higher in HRG **(E,K)**. The Kaplan-Meier curve revealed reduced survival in HRG (p < 0.001) **(F,L)**. ROC analysis confirmed the model’s predictive validity for one-, three-, and 5-year survival (AUC >0.6) **(G,M)**. The heat map showed elevated F2 and KYNU expression in HRG **(H,N)**.

Cox regression analysis was performed on 517 LUAD samples from TCGA-LUAD to pinpoint genes significantly associated with overall survival. ALOX5AP, CRP, F2, GPX3, KMO, KYNU, SOD3, and TXNRD1 were ultimately retained in the analysis; among these, ALOX5AP, CRP, and F2 were identified as risk factors for LUAD (HR > 1) (Figure 2A). The results of the proportional hazards assumption test were illustrated using Schoenfeld residual plots; the trend lines of the residuals did not show significant differences from one another (p > 0.05) (Supplementary Figure S1A–H). Subsequently, a multivariate Cox regression model was constructed that ultimately screened four prognostic genes: F2, GPX3, KMO, and KYNU (Figure 2B). The overall proportional hazards assumption test for this model yielded p = 0.228, indicating compliance with the proportional risk assumption (Supplementary Figure S1I) (Supplementary Table S5). Risk scores were determined using the coefficients associated with these prognostic genes. As a result, the 517 LUAD patients were divided into a HRG (n = 57) and a LRG (n = 460), based on an optimal cut-off value of 1.705 (Figure 2C). Principal component analysis demonstrated distinct separation between PC1 and PC2 for HRG and LRG groups (Figure 2D).

Conversely, mortality rates were found to be significantly higher within the HRG compared to LRG individuals (Figure 2E). The Kaplan-Meier curve revealed substantial disparities in survival rates between groups; those categorized as high-risk exhibited markedly reduced survival probabilities (p < 0.001) (Figure 2F). Furthermore, the ROC assessment of the risk model validated its effectiveness in forecasting survival probabilities at 1-, 3-, and 5-year for LUAD patients (AUC >0.6) (Figure 2G). The heat map illustrating expression levels of prognostic genes indicated that F2 and KYNU had elevated expression levels in HRG compared to other groups (Figure 2H).

To assess the stability of the risk model, the same set of analyses were carried out using the dataset of the validation set. The obtained results were in line with those from the TCGA-LUAD. This consistency effectively demonstrated that the prognostic model developed in this research is capable of being utilized to predict the prognosis of patients afflicted with LUAD (Figures 2I–N).

### 3.4 Establishment of the nomogram

**FIGURE 3:**
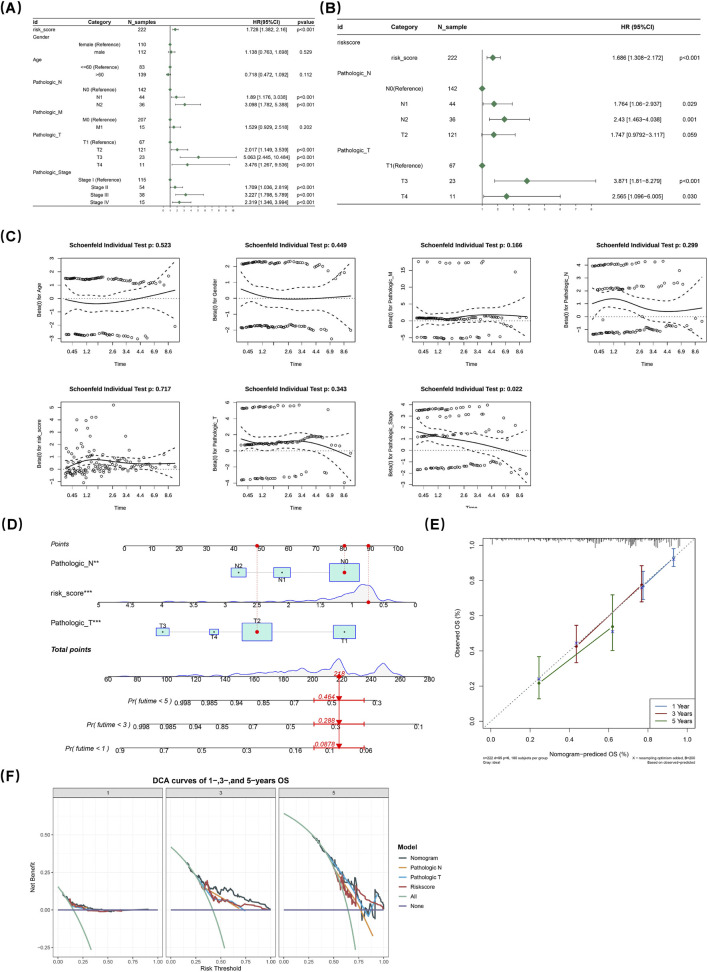
A comprehensive univariate Cox regression analysis examined the impact of individual factors on prognosis, revealing significant associations between specific factors and adverse outcomes. A proportional hazards assumption test (p > 0.05) confirmed the reliability of the results. Three variables, risk assessment score, pathologic T stage, and pathologic N stage, were identified as potential prognostic indicators **(A)**. Subsequent multivariate analysis (p < 0.05) underscored their independent prognostic significance, further validated by another proportional hazards test (p > 0.05) **(B,C)**. The nomogram indicated higher total points correlated with poorer survival in LUAD patients **(D)**. A calibration plot showed excellent model fit at 1, 3, and 5 years post-diagnosis **(E)**. Decision curve analysis demonstrated the nomogram’s net benefit exceeded zero and outperformed other options **(F)**.

A comprehensive univariate Cox proportional hazards regression analysis was systematically conducted to examine the impact of individual factors on prognosis. The analyses revealed a significant association between specific factors and adverse clinical outcomes (HR < 1, P < 0.05). Concurrently, a proportional hazards assumption test was performed, with the resulting p-value exceeding 0.05 indicating that the data met the proportional hazards assumption, thereby ensuring the reliability and validity of the univariate analysis results. Following this thorough evaluation, three variables, namely, risk assessment score, pathologic T stage, and pathologic N stage, were identified as potential prognostic indicators (Figure 3A).

Further multivariate Cox regression analyses were conducted to assess their independent predictive value. The multivariate analysis (p < 0.05) provided statistical evidence underscoring the importance of these variables. Additionally, another proportional hazards assumption test further validated the appropriateness of the model (p > 0.05). Collectively, these analyses robustly confirmed that risk score, pathologic T stage, and pathologic N stage are independent prognostic indicators (Figures 3B,C).

The nomogram illustrated that higher total points corresponded to poorer survival likelihood in LUAD patients (Figure 3D). A calibration plot was employed to assess the predictive accuracy of the nomogram; notably, at time points of 1-, 3-, and 5 years post-diagnosis, the slopes of these calibration plots closely aligned with reference lines, indicating an excellent fit for our model (Figure 3E). Furthermore, decision curve analysis curves demonstrated that the net benefit derived from using this nomogram exceeded zero and surpassed those associated with “all” or “none” options while generally outperforming both pathologic T/N staging and risk scores alone (Figure 3F).

### 3.5 Assessment of clinical features

**FIGURE 4:**
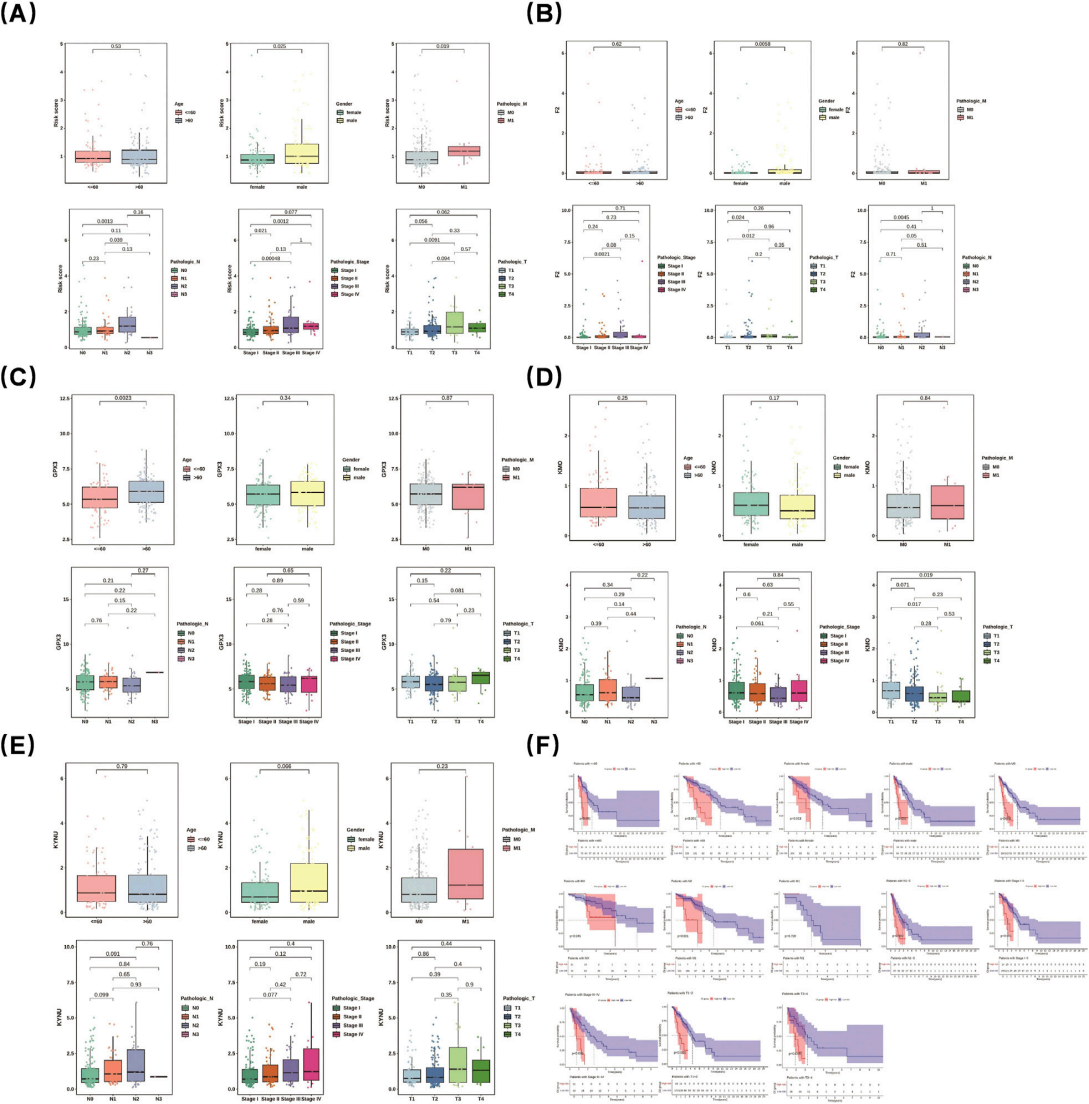
Risk scores varied across clinical features. Male patients had significantly higher risk scores than females. Box plots showed significant differences in risk scores among subgroups **(A)**. Prognostic gene analysis revealed GPX3 and KYNU were more highly expressed in males, while KMO and F2 showed the opposite. KYNU expression increased in stage IV LUAD **(B–E)**. Among stage III-IV LUAD patients, survival disparities existed, notably in pathologic N0 and T1-2 patients across age groups (>60 vs. ≤ 60 years) **(F)**.

Differences in risk scores were observed across various clinical features. Specifically, male patients exhibited a significantly higher risk score than female patients (p = 0.0058). Notably, box plots demonstrated significant differences in risk scores among different subgroups of clinical features (T1 and T3, stage I and stage III, N1 and N2, stage I and stage II, M0 and M1, N0 and N2, as well as between stages I and IV) (p < 0.05) (Figure 4A). Further analysis of prognostic genes within distinct clinical subgroups revealed that GPX3 and KYNU exhibited higher expression levels in male patients than in female patients; conversely, KMO and F2 showed the opposite trend. Additionally, the expression of KYNU displayed an increasing trend in cases of stage IV LUAD (Figures 4B–E). Remarkably, among LUAD patients at stages III-IV, significant survival disparities persisted between two groups. This was particularly evident among patients with pathologic N0 status as well as those with pathologic T1-2 status across all age groups (>60 years old vs. ≤ 60 years old) (p < 0.001) (Figure 4F).

### 3.6 GSEA enrichment analysis of DEGs

**FIGURE 5:**
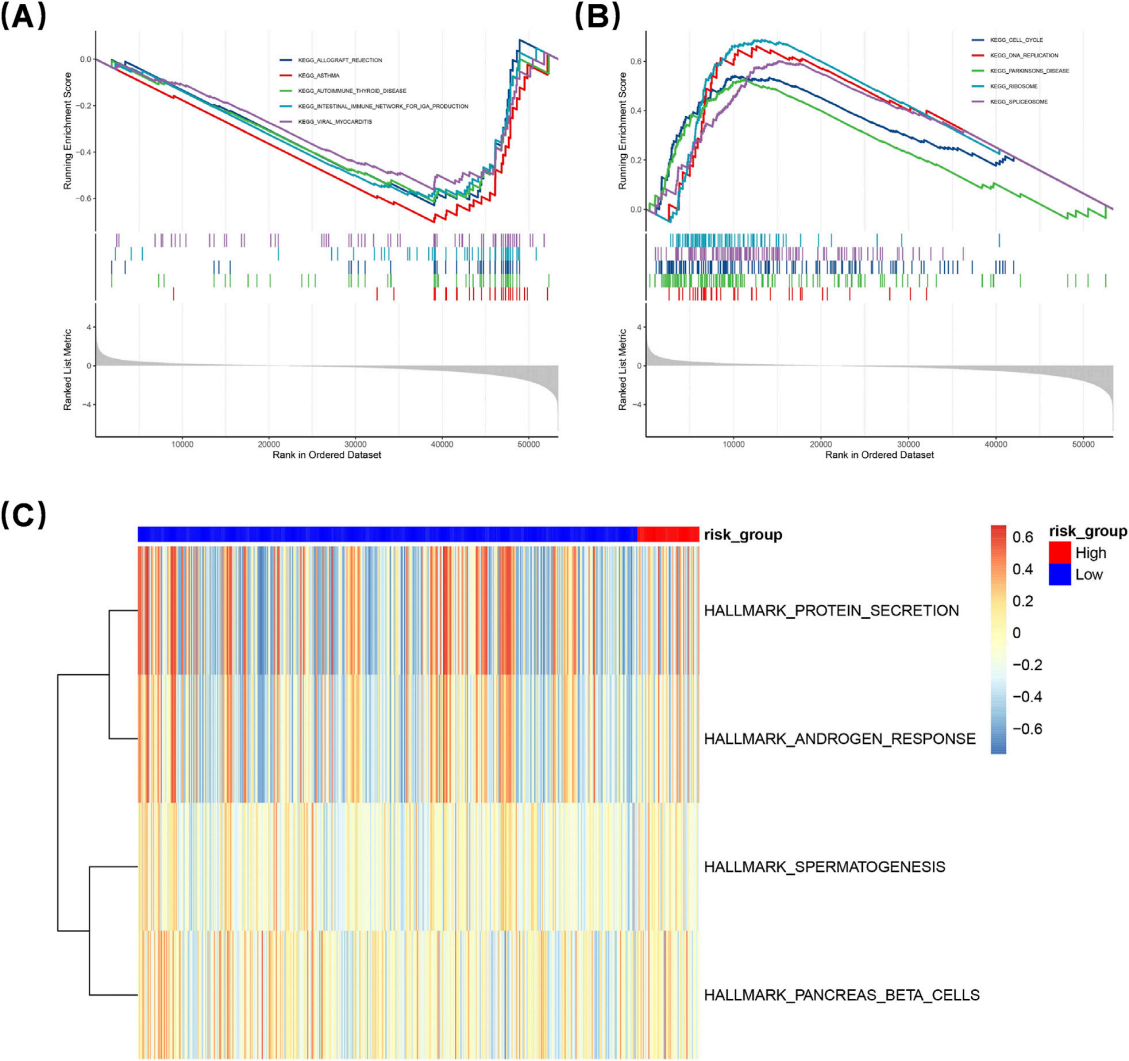
Gene Set Enrichment Analysis (GSEA) was conducted to elucidate gene expression differences. The low-risk group (LRG) showed activation of immune-related pathways like autoimmune thyroid disease and asthma. The high-risk group (HRG) exhibited enrichment in pathways linked to cell proliferation, metabolism, and gene regulation, including DNA replication and cell cycle progression **(A,B)**. Gene Set Variation Analysis (GSVA) further assessed pathway activation **(C)**. HRG pathways, with core genes like MCM7 and PCNA, suggest enhanced cell proliferation. Genes like CDK6 and CCNB1 also support HRG’s role in promoting cellular proliferation.

To elucidate the signaling mechanisms underlying the DEGs between the two groups, GSEA was performed. A total of 50 pathway entries were identified through this analysis. Notably, in the LRG, several pathways associated with immune modulation and disorders were activated, including autoimmune thyroid disease, allograft rejection, asthma, and the intestinal immune network for IgA production. In contrast, the high-risk Group (HRG) exhibited significant enrichment in multiple pathways related to cell proliferation, metabolism, and gene regulation, specifically DNA replication, cell cycle progression, spliceosome activity, and ribosomal function (Figures 5A,B). Subsequently, GSVA was employed to further evaluate the activation or inhibition status of these pathways within both groups (Figure 5C). In particular, certain pathways in the HRG showed notable enrichment that suggests their critical role in intestinal immunomodulation. Core genes within these enriched pathways in the HRG, including MCM7, PCNA, and POLE, are recognized as essential players in DNA replication and repair processes. This observation implies a likely enhancement of cell proliferation activities within the HRG. Furthermore, genes such as CDK6, CCNB1, and CDK1 are known regulators of cell cycle progression; this finding further substantiates that the HRG promotes cellular proliferation.

### 3.7 Tumor microenvironment characterization

**FIGURE 6:**
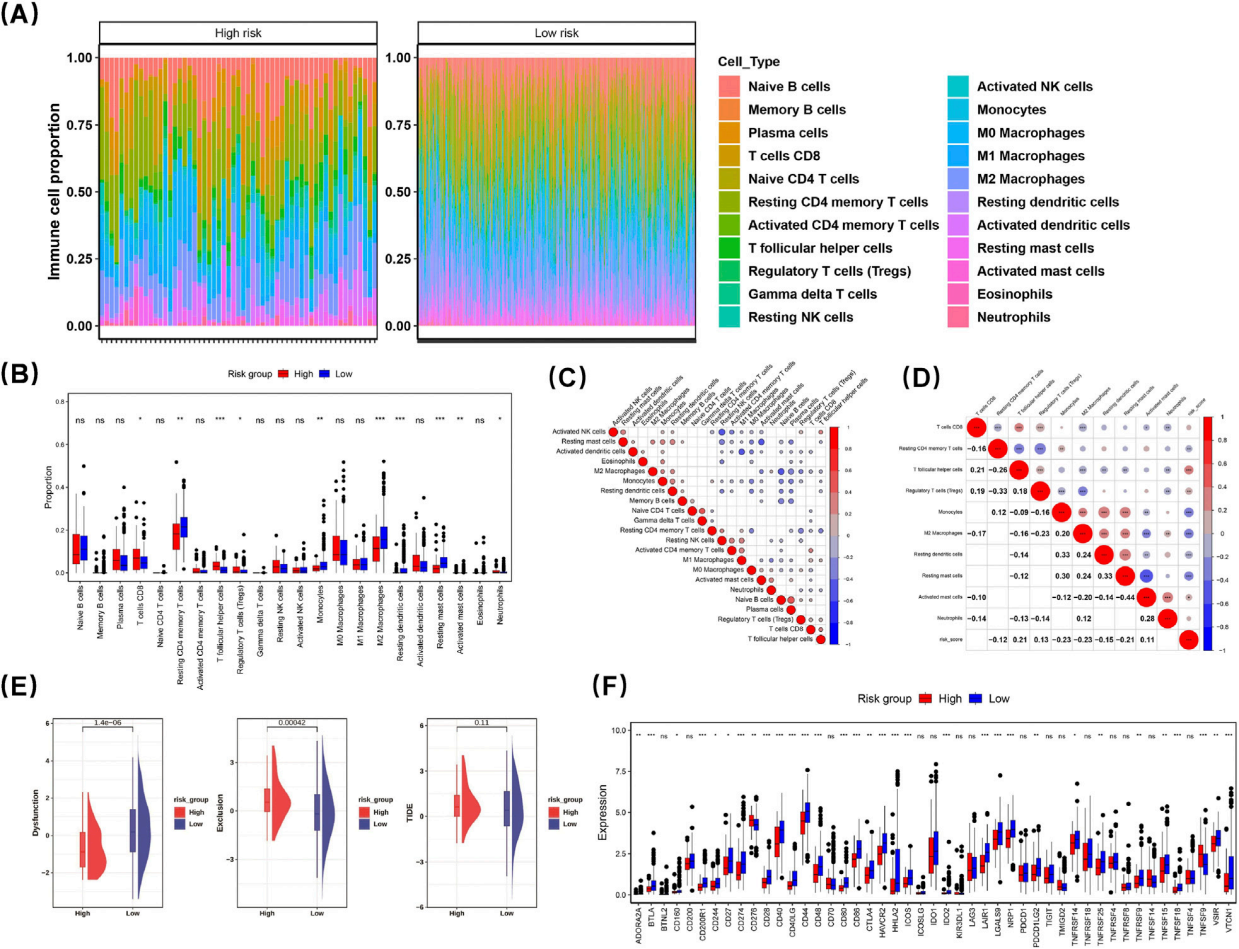
Characterization of LUAD tumor microenvironment. A heatmap was generated to show the enrichment ratios of 22 immune cell subtypes across risk levels **(A)**. Immune cells with differing infiltration levels between groups were deemed differentially infiltrating. A box-and-whisker plot depicted their percentages in both groups, revealing significant differences in ten immune cell types, including CD8^+^ T cells and regulatory T cells (p < 0.05) **(B)**. Spearman’s rank correlation analysis showed plasma cells and naive B cells had a strong positive correlation (cor = 0.5825, p < 0.001), while resting NK cells and activated NK cells showed a strong negative correlation (cor = −0.4887, p < 0.001) (**(C)**, Supplementary Figure S1). A heatmap indicated resting dendritic cells and resting mast cells positively correlated with monocytes (cor = 0.33; cor = 0.30; p < 0.05), whereas activated mast cells negatively correlated with resting mast cells (cor = −0.44; p < 0.05) **(D)**. TIDE analysis revealed the dysfunction indicator in the high-risk group (HRG) was lower than in the low-risk group (LRG), suggesting higher immune escape likelihood, while Exclusion was greater in HRG, indicating reduced immune escape probability **(E)**. Among 47 immune checkpoint genes, 32 showed differential expression across risk groups (p < 0.05) **(F)**. CD276 and TNFSF4 were elevated in HRG, while 30 genes were higher in LRG.

A heatmap was generated to illustrate the enrichment ratios of 22 distinct immune cell subtypes across various risk levels (Figure 6A). Immune cells exhibiting diverse infiltration levels between the two groups were categorized as differentially infiltrating immune cells. Subsequently, a box-and-whisker plot was utilized to depict the percentage of these differentially infiltrating immune cells in both groups. Significant disparities were observed in the infiltration percentages of ten types of immune cells between the two groups, including CD8^+^ T cells and resting memory CD4^+^ T cells (p < 0.05) (Figure 6B).

Further analysis using Spearman’s rank correlation revealed that plasma cells and naive B cells exhibited a robust positive correlation (cor = 0.5825, p < 0.001), while resting NK cells demonstrated a strong negative correlation with activated NK cells (cor = −0.4887, p < 0.001) (Figure 6C) (Supplementary Figure S1). Notably, a heatmap illustrating the correlation between risk scores and differential immune cell populations indicated that both resting dendritic cells and resting mast cells had positive correlations with monocytes (cor = 0.33; cor = 0.30; p < 0.05). In contrast, activated mast cells showed a negative correlation with resting mast cells (cor = −0.44; p < 0.05) (Figure 6D). This finding suggests that expression levels of prognostic genes are closely associated with the presence of resting mast cells, indicating that these genes may play a significant role in this relationship. These results could enhance our understanding of disease prognosis and underlying immunological mechanisms.

TIDE analysis showed that the dysfunction indicator in the HRG was notably reduced compared with the LRG, suggesting a greater potential for immune escape. Conversely, the value of Exclusion in the HRG was markedly greater than that in the LRG, suggesting a reduced probability of immune escape (Figure 6E). The immune checkpoint mechanism plays a crucial role in evading detection and attack by T cells. We examined and compared the expression patterns of genes associated with immune checkpoints across different risk categories. Among the 47 immune checkpoint genes analyzed, 32 exhibited differential expression among distinct risk groups (p < 0.05) (Figure 6F). Notably, CD276 and TNFSF4 showed elevated expression levels in the HRG compared to those in the LRG. In contrast, the remaining 30 genes demonstrated higher expression levels within the LRG when compared to those in the HRG. The varying expressions of these genes provide a foundation for categorizing LUAD patients who may be suitable candidates for immunosuppressive treatment.

### 3.8 Examination of somatic cell mutations and assessment of drug sensitivity

**FIGURE 7:**
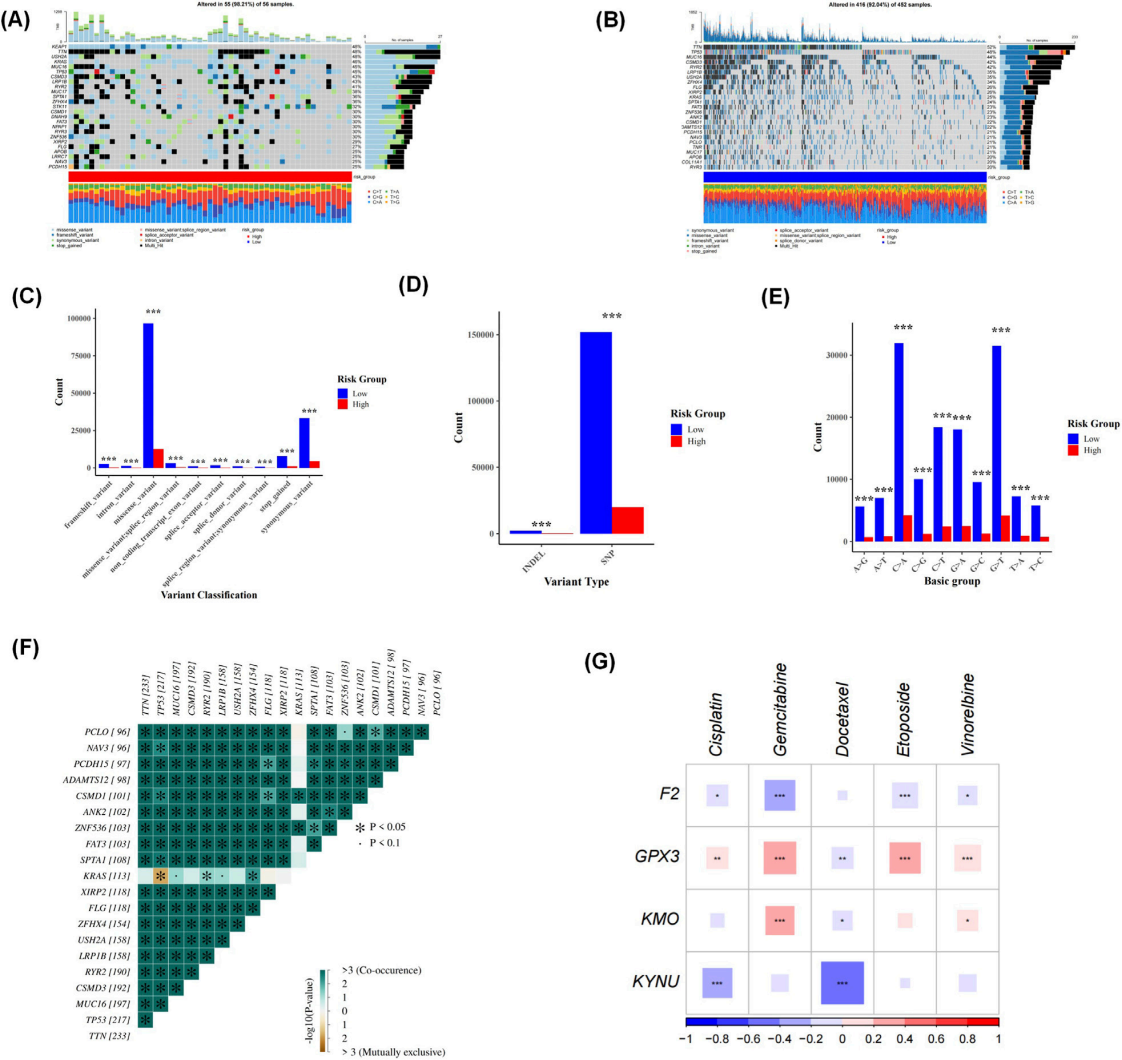
Somatic mutation analysis showed a higher mutation percentage in the high-risk group (HRG) than in the low-risk group (LRG). KEAP1, TTN, and USH2A had the highest mutation rates in HRG (48%), while TTN showed the highest rate in LRG (52%), suggesting TTN is frequently mutated in lung adenocarcinoma (LUAD) patients **(A,B)**. Detailed analysis revealed that synonymous and missense variants, along with single nucleotide polymorphisms (SNPs), were more common in LRG. G > T and C > A base changes were also more prevalent in LRG **(C–E)**. Drug sensitivity analysis identified 124 compounds with differential IC50 values between the two groups (Supplementary Table S7). The analysis of the mutual exclusivity or co-occurrence of mutations revealed that several key genes showed significant interrelationships in the lung adenocarcinoma cohort. **(F)**. Five first-line LUAD chemotherapeutic agents were highlighted: cisplatin, gemcitabine, docetaxel, etoposide, and vinorelbine. A significant correlation was found between KUNU and Docetaxel (p < 0.01) **(G)**.

The results from the somatic mutation analysis revealed a higher mutation percentage in the HRG compared to the LRG) with rates of 98.21% versus 92.94%, respectively. Notably, KEAP1, TTN, and USH2A exhibited the highest mutation rates within the HRG at 48%, while TTN displayed the highest mutation rate in the LRG at 52%. This suggests that TTN may be one of the genes most frequently mutated in patients with LUAD (Figures 7A,B). A detailed analysis of the mutation data indicated that synonymous variants and missense variants occurred more frequently in the LRG. Furthermore, single nucleotide polymorphisms (SNPs) were also more prevalent in this group. Interestingly, G > T and C > A base changes were observed to be more common in the LRG as well. These mutations showed statistical significance between HRG and LRG. (Figures 7C–E). In addition, most of the top 20 mutated genes showed significant co-occurrence, and in particular, KRAS and TP53 exhibited mutual exclusivity (p < 0.05) (Figure 7F).

Moreover, drug sensitivity analysis identified a total of 124 compounds exhibiting differential IC50 values between these two groups (Supplementary Table S7). From this screening process, five first-line chemotherapeutic agents for LUAD treatment were highlighted: cisplatin, gemcitabine, docetaxel, etoposide, and vinorelbine. The findings demonstrated a significant correlation between KYNU and Docetaxel (p < 0.01) (Figure 7G). The respective IC50 values for these drugs were lower in the HRG cohort, indicating that individuals within this group exhibited greater sensitivity to these therapeutic agents. Additionally, further confirmation was provided by observing that IC50 values for these drugs remained consistently lower among those classified within HRG; thus reinforcing their heightened responsiveness to such treatments.

### 3.9 Expression validation

**FIGURE 8:**
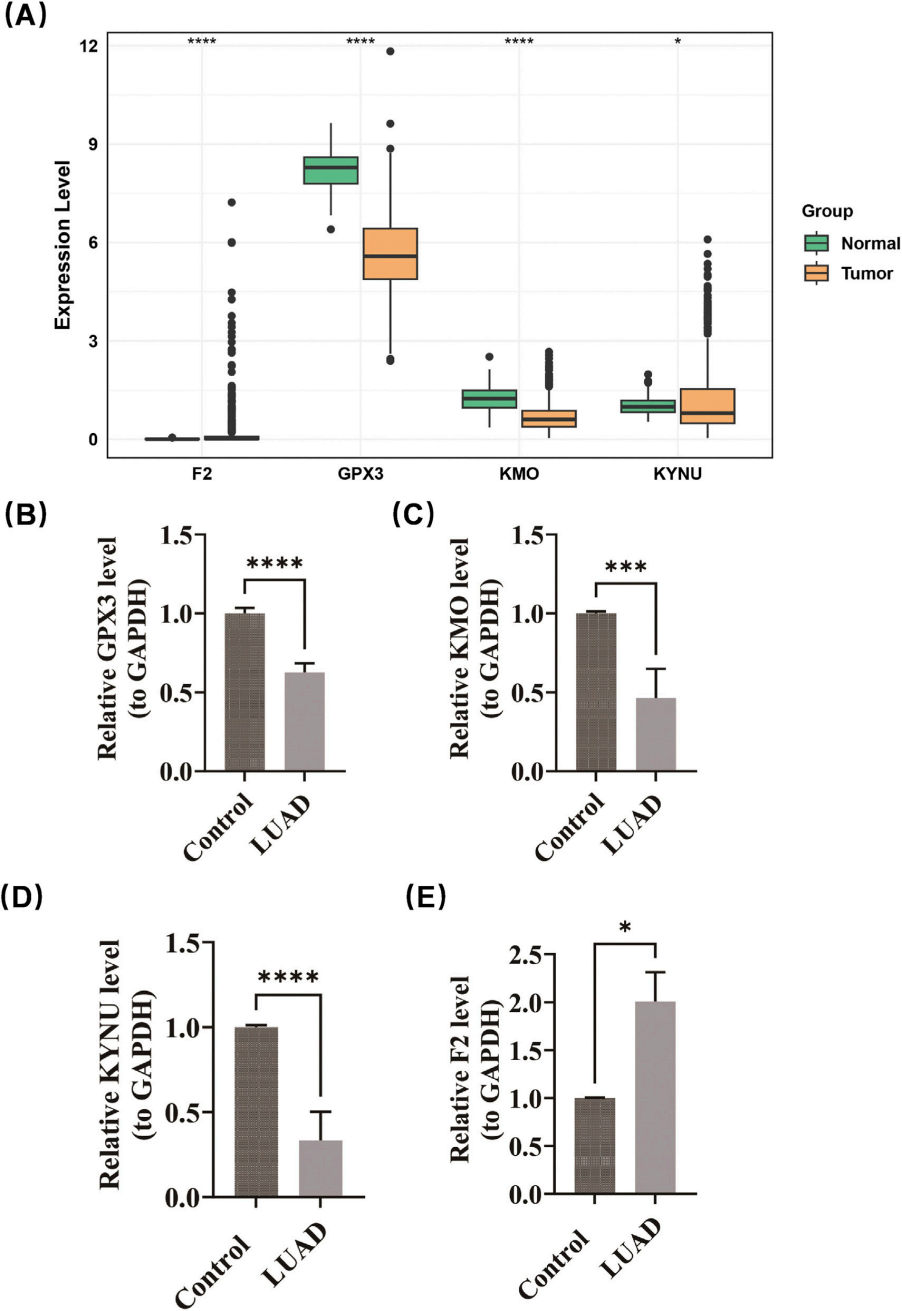
Expression validation confirmed the differential patterns of prognostic genes. GPX3 (p < 0.0001), KYNU (p < 0.05), and KMO (p < 0.0001) were found to be downregulated in LUAD, while F2 exhibited significant upregulation (p < 0.0001) **(A)**. RT-PCR demonstrated that GPX3, KMO, and KYNU were significantly downregulated in LUAD, whereas F2 was upregulated in this context **(B–E)**.

Expression validation confirmed the differential patterns of prognostic genes. Compared to normal samples, GPX3 (p < 0.0001), KYNU (p < 0.05), and KMO (p < 0.0001) were found to be downregulated in LUAD, while F2 exhibited significant upregulation (p < 0.0001) (Figure 8A). The experimental results obtained from RT-PCR demonstrated a high degree of consistency with the predictions derived from bioinformatics analyses. In comparison to normal samples, GPX3, KMO, and KYNU were significantly downregulated in LUAD, whereas F2 was upregulated in this context (Figures 8B–E). The amplification curves (obtained after 20 cycles) and melting curves (showing smooth, single peaks) demonstrated good primer specificity and favorable amplification efficiency (Supplementary Figure S2). These findings are consistent with expression profiles derived from TCGA, thereby reinforcing the biological relevance of the prognostic signature.

## 4 Discussion

Lung adenocarcinoma (LUAD), the predominant subtype of non-small cell lung cancer, continues to present significant challenges due to its heterogeneity and resistance to treatment (Zheng et al., 2025). Therefore, it is crucial to promptly identify patients at risk for recurrence and develop personalized treatment strategies tailored to their needs. As a trace element, selenium plays complex regulatory roles in various immune cells. In recent years, there has been growing interest in the role of selenium metabolism-related genes (SMRGs) in modulating tumor oxidative stress and reshaping the immune microenvironment. However, their prognostic significance and underlying molecular mechanisms remain largely unexplored (Fu et al., 2023; Ahmed et al., 2025). This study employed bioinformatics analysis to establish a risk model based on selenium metabolism in LUAD and further examined its relationships with the tumor microenvironment, somatic mutations, and drug sensitivity. These findings enhance our understanding of LUAD progression and provide valuable insights for developing more precise therapeutic strategies.

In this research, we identified four hub genes associated with selenium metabolism through regression analysis. Additionally, we developed a risk model for calculating the selenium metabolism score. The four SMRGs identified in this study represent potential vulnerabilities in cancer cells, providing functional targets for novel therapies against LUAD. GPX3, a member of the glutathione peroxidase family, inhibits ferroptosis by neutralizing lipid peroxides. Its downregulation is correlated with poor survival outcomes in high-risk patients characterized by elevated tumor mutational burden (TMB) and KEAP1 mutation enrichment (Shimada et al., 2022; Metlay et al., 1995; Zhang F. et al., 2025). Enrichment analysis revealed that GPX3 is significantly associated with antioxidant and peroxidase activities, suggesting that dysregulated oxidative stress contributes to tumor progression. Studies indicate that GPX3 may serve as a diagnostic biomarker for oxidative stress-induced encephalitis; furthermore, GPX4, another family member, regulates reactive oxygen species (ROS) levels in breast cancer cells to resist ferroptosis (Lee et al., 2021). In lung cancer specifically, GPX3 expression is silenced via methylation, a phenomenon linked to metastasis and chemotherapy resistance in LUAD. It is plausible that GPX3 expression correlates with clinical indicators such as disease stage and prognosis. Moreover, GPX3 emerges as a promising prognostic marker and therapeutic target for both LUAD and lung squamous cell carcinoma (LUSC) (Zhang et al., 2025b; Chełchowska et al., 2025). KMO functions as kynurenine 3-monooxygenase and plays a pivotal role in the kynurenine metabolic pathway by catalyzing the conversion of kynurenine to 3-hydroxykynurenine. This pathway has been implicated in inflammation, oxidative stress, and neurotoxicity (Zhang M. et al., 2025). Aberrant expression of KMO in tumors may significantly influence immune regulation within the tumor microenvironment. Research indicates that alterations in the kynurenine pathway are associated with mechanisms of immune evasion in cancer cells. Elevated KMO expression may modulate immune cell activity by affecting metabolite levels, thereby impacting patient outcomes (Chen et al., 2025). In stroke research, KMO has been shown to inhibit mitochondrial autophagy, facilitating brain repair following a stroke (Wang et al., 2024). The differential expression of KMO between high- and low-risk groups in this study may correlate with adverse prognoses; furthermore, KMO has been identified as a protective factor against recurrence in LUAD. Investigating compounds involved in selenium metabolism could elucidate the role of KMO in tumor immune escape and disease progression, potentially identifying it as a target for combinational therapy. KYNU, also known as kynureninase, is a key enzyme within the kynurenine pathway responsible for converting kynurenine into anthranilic acid. Dysregulation of KYNU has been observed across various cancers and is closely linked to tumor initiation and progression (Xu et al., 2025). Its metabolites can influence cellular redox states, thereby affecting both tumor cell proliferation and apoptosis (Zhang et al., 2025d). Additionally, changes in KYNU activity may impair immune cell function, contributing to tumor immune escape (Xiang et al., 2024). Within the risk model developed in this study, variations in KYNU expression may serve as critical determinants of patient prognosis. Exploring upstream and downstream regulatory pathways related to KYNU could uncover new therapeutic avenues for treating LUAD. The F2 gene encodes coagulation factor II (prothrombin), which plays a central role in the coagulation cascade. Beyond its classical function in hemostasis, emerging evidence links abnormalities involving coagulation factor II and related pathways to tumor metastasis (Teofilov et al., 2025), angiogenesis (Kvasnička et al., 2024), and disease severity (PMID: 39497411). Activation of the coagulation system by tumor cells facilitates angiogenesis and metastasis. Altered expression of F2 may influence these processes, potentially elucidating the poorer outcomes observed in high-risk LUAD patients. For example, F2 may play a role in microthrombosis formation within tumors or regulate factors that drive angiogenesis. Clinically, evaluating coagulation parameters such as D-dimer levels in LUAD patients could provide insights into whether anticoagulant therapy enhances prognosis.

Subsequently, we undertook a more comprehensive exploration of the molecular characteristics associated with distinct risk populations through GSEA. Pathway enrichment analysis in the high-risk group revealed significant involvement of pathways related to cell proliferation, metabolism, and gene regulation. Notably, these included DNA replication, the cell cycle, spliceosome activity, and ribosomal pathways. These findings are consistent with previous studies that have documented uncontrolled cell cycle progression and aberrant DNA replication in LUAD (Wu et al., 2025). In contrast, the low-risk group demonstrated an enrichment of immune-related pathways. These encompassed those linked to asthma, autoimmune thyroid disease, transplant rejection, and immunoglobulin A (IgA) production within the intestinal immune network. Selenium intake has been associated with asthma prevalence (Zajac, 2021) and plays a role in selenium metabolism. Furthermore, serum selenium levels correlate with thyroid disorders; specifically, selenium deficiency is known to elevate the risk of autoimmune thyroid conditions (Troshina et al., 2024).

Immune cell infiltration analysis revealed an increased proportion of M2 macrophages and resting CD4 memory T cells in high-risk tumors, whereas low-risk tumors exhibited elevated levels of CD8^+^ T cells and resting dendritic cells. These findings corroborate previous studies regarding the roles of immune cells within the tumor microenvironment. M2 macrophages and neutrophils are known to promote tumor growth and facilitate immune evasion (Zeng et al., 2025), while CD8^+^ T cells and dendritic cells are pivotal in driving anti-tumor immunity. The differential expression of immune checkpoint genes across risk groups indicates varying efficacies of immunotherapy (Jiang et al., 2025). In the realm of cancer immunity, selenium has been shown to enhance lysosomal activity and cytotoxicity in CD8^+^ T cells (Chen et al., 2019). These results underscore the significance of the immune microenvironment in LUAD progression and advocate for personalized immunotherapeutic strategies. The development of selenium nanoparticles or selenium-containing compounds may improve selenium protein status in LUAD cells, thereby synergistically enhancing the efficacy of immunotherapy.

For LUAD cases that lack driver gene mutations, chemotherapy remains the primary treatment option, often in combination with immunotherapy or anti-angiogenic agents. The first-line regimen typically consists of platinum-based drugs alongside pemetrexed. In this study, we analyzed the differences in chemosensitivity between high-risk and low-risk LUAD patients. Notably, cisplatin demonstrated significantly lower IC50 values in the high-risk group compared to the low-risk group, indicating enhanced efficacy among high-risk patients. Cisplatin induces cytotoxicity by damaging tumor cell DNA and activates the immune system through immunogenic cell death (ICD). In high-risk patients, cisplatin may enhance antigen presentation by dendritic cells via the release of immune-stimulatory molecules such as calreticulin (CRT) and HMGB1, thereby activating T-cell-mediated anti-tumor responses (Ma et al., 2025; Yasuda et al., 2025). Similarly, docetaxel, gemcitabine, and etoposide exhibit superior efficacy in high-risk patients; this is likely attributable to their ability to inhibit tumor angiogenesis, reverse epithelial-mesenchymal transition (EMT), and exert immunomodulatory effects such as reducing Treg activity (Massa et al., 2025; Obradovic et al., 2023) while promoting M1 polarization of tumor-associated macrophages (TAMs) (Jiménez-Cortegana et al., 2021). These agents also regulate the tumor microenvironment to suppress angiogenesis (Al-Omar et al., 2025), providing a rationale for combining chemotherapy with immunotherapy in high-risk LUAD patients. Our analysis of prognostic gene-drug associations revealed that GPX3 expression significantly correlates with drug IC50 values. It is crucial to conduct further research on how GPX3 interacts with selenium metabolism and chemotherapy drugs to influence both the occurrence and progression of LUAD.

This project systematically evaluated the pivotal role of SMRGs in LUAD by integrating transcriptome data analysis. We developed a risk score model based on these genes to distinguish high-risk patients and predict their prognosis. At the mRNA level, we conducted a preliminary validation of the four prognostic genes through RT-qPCR in five pairs of LUAD and adjacent non-tumor tissues. The expression difference patterns of the genes were basically consistent with the trends of the TCGA-LUAD data. However, it should be noted that the current validation only focused on the “expression differences of the genes between tumor and normal tissues”,and could only serve as preliminary exploratory evidence that the genes screened out by bioinformatics have real expression differences. The results suggest that patients in the high-risk group may experience a more complex immunosuppressive microenvironment. Future research could further explore the related pathways enriched by these core genes through cell and animal experiments to elucidate their specific mechanisms in tumor progression and immune evasion. Additionally, these genes hold potential as diagnostic and therapeutic targets, which may facilitate the development of novel selenium metabolism therapies or precise nutritional supply models, thereby providing new avenues for personalized treatment and precision medicine in LUAD. However, certain limitations must be acknowledged. Firstly, this study mainly relies on bioinformatics analysis of public datasets. There are two major deficiencies in the existing experimental validation: on the one hand, the expression of prognostic genes was only verified through RT-qPCR experiments on five pairs of LUAD and adjacent non-tumor tissues. Although the trend is consistent with the TCGA dataset and the authenticity of gene expression was preliminarily explored, the clinical application value of the prognostic model was not touched upon. On the other hand, due to the limitations of clinical sample acquisition conditions, the current validation sample size is only five pairs. Although technical repetition has ensured the reliability of the results, the small sample size may still lead to insufficient statistical power and cannot be widely promoted. Secondly, there is a lack of *in vitro* and *in vivo* functional validation experiments, which makes the mechanism explanation of the model insufficient. In addition, as this study is a retrospective study, bias may be difficult to avoid; therefore, it is necessary to further verify the findings of this study through prospective studies.

## 5 Conclusion

In conclusion, we have developed a risk model associated with selenium metabolism genes to predict recurrence in patients diagnosed with LUAD. The accuracy of this model was further validated using an external validation cohort. This signature demonstrates a robust prognostic predictive capability and can be utilized to characterize the tumor microenvironment of LUAD. The novel methodologies and key genes identified in our study may offer valuable insights for advancing precision oncology in LUAD.

## Data Availability

The datasets presented in this study can be found in online repositories. The names of the repository/repositories and accession number(s) can be found in the article/Supplementary Material.

## Supplementary material

Supplementary Figure S1 The results of the proportional hazards assumption test were illustrated using Schoenfeld residual plots. The trend lines of the residuals did not show significant differences from one another (p > 0.05) **(A–H)**. The overall proportional hazards assumption test for this model yielded p = 0.228, indicating compliance with the proportional risk assumption **(I)**.

Supplementary Figure S2 The amplification curves and melting curves for significant genes identified in this study. **(A–E)** present the amplification curvers for F2 **(A)**, GPX3 **(B)**, KMO **(C)**, KYNU **(D)** and GAPDH **(E)**, respectively. **(F–J)** present the milting curvers for F2 **(F)**, GPX3 **(G)**, KMO **(H)**, KYNU **(I)** and GAPDH **(J)**, respectively.
