# Supplementary figures and images for: Identification and validation of selenium metabolism-related genes in lung adenocarcinoma prognosis using bioinformatics analysis
Source: Front Genet. 2025 Oct 16;16:1655262. doi: 10.3389/fgene.2025.1655262 (PMC12571454; doi:10.3389/fgene.2025.1655262)

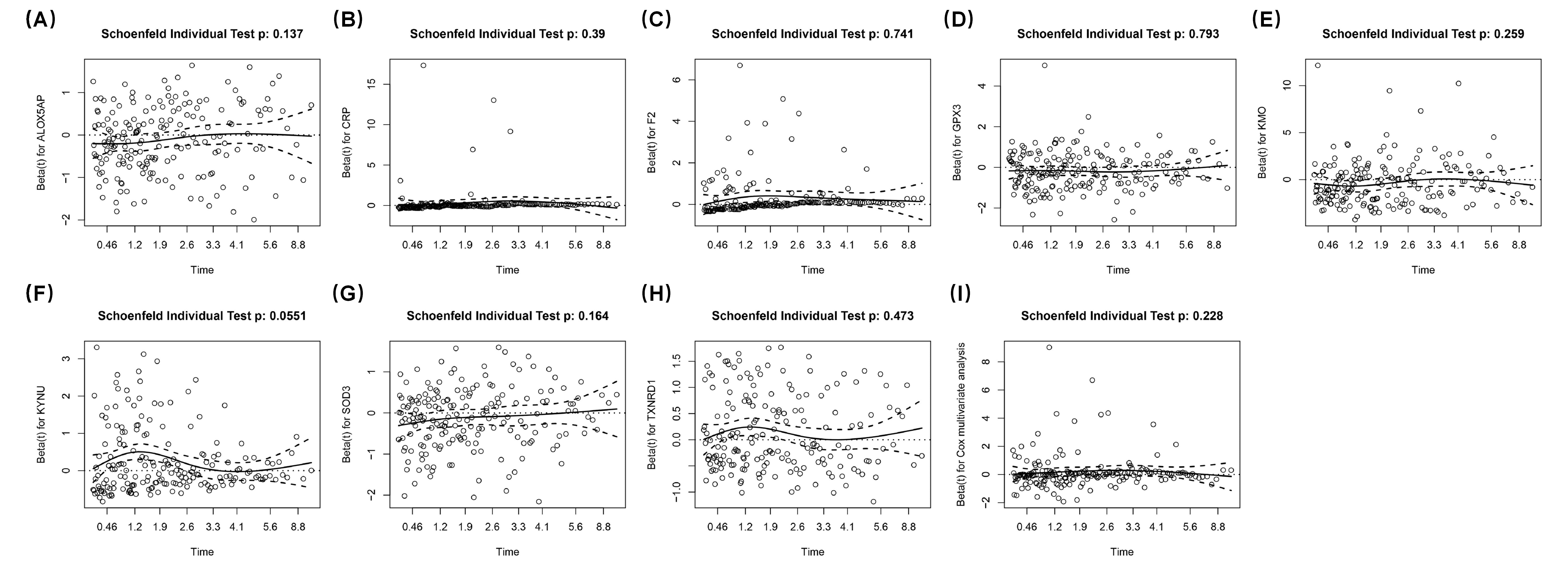

Supplement: Supplementary file 3 [file Image1.jpeg]

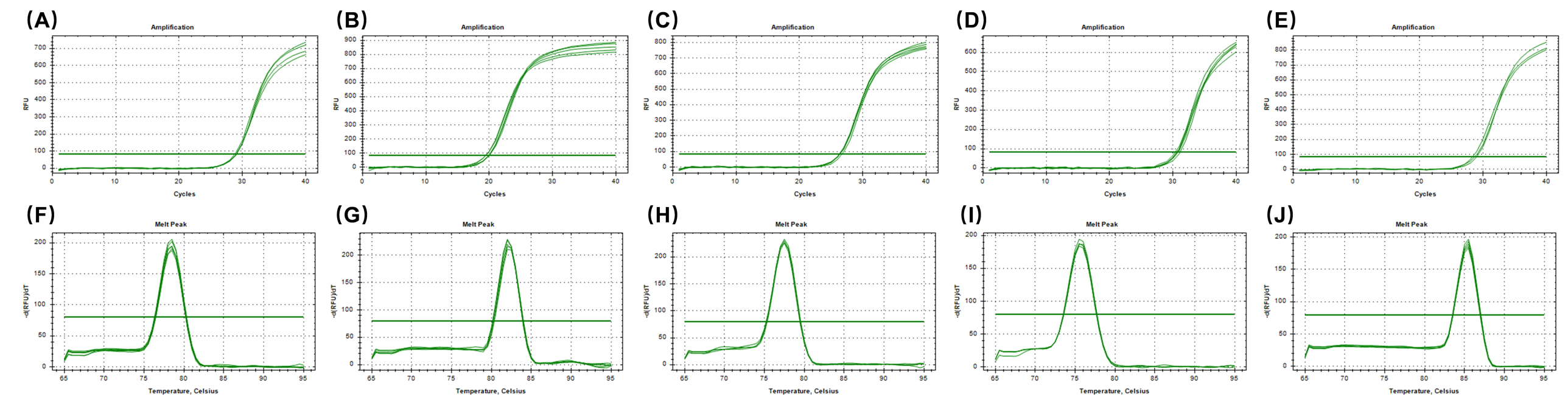

Supplement: Supplementary file 4 [file Image2.jpeg]
